# Ouabain Contributes to Kidney Damage in a Rat Model of Renal Ischemia-Reperfusion Injury

**DOI:** 10.3390/ijms17101728

**Published:** 2016-10-14

**Authors:** Luca Villa, Roberta Buono, Mara Ferrandi, Isabella Molinari, Fabio Benigni, Arianna Bettiga, Giorgia Colciago, Masami Ikehata, Elisabetta Messaggio, Maria Pia Rastaldi, Francesco Montorsi, Andrea Salonia, Paolo Manunta

**Affiliations:** 1 Division of Experimental Oncology/Unit of Urology, IRCCS San Raffaele Scientific Institute, 20132 Milan, Italy; villa.luca@hsr.it (L.V.); buono@usc.edu (R.B.); benigni.fabio@hsr.it (F.B.); bettiga.arianna@hsr.it (A.B.); colciago.giorgia@hsr.it (G.C.); montorsi.francesco@hsr.it (F.M.); salonia.andrea@hsr.it (A.S.); 2 Prassis Sigma-Tau Research Institute, Settimo Milanese, 20019 Milan, Italy; mara.ferrandi@gmail.com (M.F.); isabella.molinari8@gmail.com (I.M.); 3 Renal Research Laboratory, Fondazione IRCCS Ca’ Granda Ospedale Maggiore Policlinico & Fondazione D’Amico, 20122 Milan, Italy; aquamarine0321@libero.it (M.I.); mariapia.rastaldi@policlinico.mi.it (M.P.R.); 4 Division of Genetics and Cell Biology, Genomics of Renal Diseases and Hypertension Unit, IRCCS San Raffaele Scientific Institute, 20132 Milan, Italy; messaggio.elisabetta@hsr.it; 5 Chair of Urology, Università Vita Salute San Raffaele, IRCCS San Raffaele Scientific Institute, 20132 Milan, Italy; 6 Chair of Nephrology, Università Vita Salute San Raffaele, IRCCS San Raffaele Scientific Institute, 20132 Milan, Italy

**Keywords:** ouabain, rostafuroxin, warm ischemia, renal damage, nephrin, Na-K ATPase

## Abstract

Warm renal ischemia performed during partial nephrectomy has been found to be associated with kidney disease. Since endogenous ouabain (EO) is a neuro-endocrine hormone involved in renal damage, we evaluated the role of EO in renal ischemia-reperfusion injury (IRI). We measured plasma and renal EO variations and markers of glomerular and tubular damage (nephrin, KIM-1, Kidney-Injury-Molecule-1, α1 Na-K ATPase) and the protective effect of the ouabain inhibitor, rostafuroxin. We studied five groups of rats: (1) normal; (2) infused for eight weeks with ouabain (30 µg/kg/day, OHR) or (3) saline; (4) ouabain; or (5) saline-infused rats orally treated with 100 µg/kg/day rostafuroxin for four weeks. In group 1, 2–3 h after IRI, EO increased in ischemic kidneys while decreased in plasma. Nephrin progressively decreased and KIM-1 mRNA increased starting from 24 h. Ouabain infusion (group 2) increased blood pressure (from 111.7 to 153.4 mmHg) and ouabain levels in plasma and kidneys. In OHR ischemic kidneys at 120 h from IRI, nephrin, and KIM-1 changes were greater than those detected in the controls infused with saline (group 3). All these changes were blunted by rostafuroxin treatment (groups 4 and 5). These findings support the role of EO in IRI and suggest that rostafuroxin pre-treatment of patients before partial nephrectomy with warm ischemia may reduce IRI, particularly in those with high EO.

## 1. Introduction

Recent findings suggest that patients with localized renal cancer are best managed with nephron-sparing surgery (partial nephrectomy) rather than radical nephrectomy [1]. However, available data show that the preservation of renal function can be hampered also in the context of conservative treatments. Prolonged intra-operative renal warm ischemia time (WIT) performed during partial nephrectomy is in fact likely to cause the development of acute renal failure in the post-operative period and the onset of chronic kidney disease throughout the follow-up in a WIT-dependent manner [2,3,4], ultimately increasing the risk of cardiovascular events and the rate of all-cause mortality [5]. Therefore, the protection against renal ischemia-reperfusion injury (IRI) is a compelling medical need and an on-going field of research aimed at preserving post-operative renal function.

To explore the molecular mechanisms underlying renal function alterations following renal warm ischemia and to assess a preventive pre-surgical treatment that might inhibit or reduce the development of subsequent renal damage, we measured well-established markers of glomerular-tubular damage, such as nephrin, KIM-1, and α1 Na-K ATPase and endogenous ouabain (EO) both in plasma and in renal tissue in an in vivo animal model of renal IRI. EO, a neuro-endocrine hormone, was measured because it is involved in the process of renal function impairment leading to tubular alterations both in rats [6] and humans [7]. EO also affects the glomerular podocyte function through the downregulation of nephrin, leading to alterations of the glomerular filtration barrier and proteinuria [6,7,8].

Recent data [8,9,10,11] show that rostafuroxin (PST2238) may antagonize the molecular and functional effects of EO and, therefore, we investigated whether rostafuroxin may induce a protective effect on IRI. Rostafuroxin is a well-characterized compound that acts by disrupting ouabain-mediated interactions, favoring the normalization of renal tubular Na-K ATPase activity linked to Src-mediated signaling pathway. Through this mechanism, rostafuroxin lowers blood pressure both in hypertensive rats [9] and in patients carrying genetic variants for adducin and EO metabolic enzymes [10]. More recently, rostafuroxin has been shown to protect from podocyte damage and proteinuria induced by ouabain and mutant adducin in animal models [8]. Therefore, rostafuroxin may provide a further proof of the role of EO in IRI and a valuable tool to prevent IRI in patients, if pre-treated before partial nephrectomy.

In the present study, we set up a rat model of unilateral nephrectomy and ischemia, to study, in different groups of rats, both the effects of ouabain and its blockade on the onset of secondary renal alterations. The results suggest that EO/ouabain, both at normal and high plasma concentrations, may be directly implicated in the worsening of renal damage, resulting from the ischemia-reperfusion maneuver, and that rostafuroxin treatment may exert a protective effect. These data may be clinically translated, particularly in patients with high plasma EO, where the development of IRI following partial nephrectomy with warm ischemia may be prevented by a pre-operative medical treatment with rostafuroxin.

## 2. Results

### 2.1. Time Course of EO and Renal Biomarker Changes after IRI (Ischemia-Reperfusion Injury)

To investigate whether IRI induced significant modifications of EO and renal biomarkers, rats were subjected to IRI and plasma and kidneys were taken after 1 to 120 h from injury (group 1).

**Figure 1:**
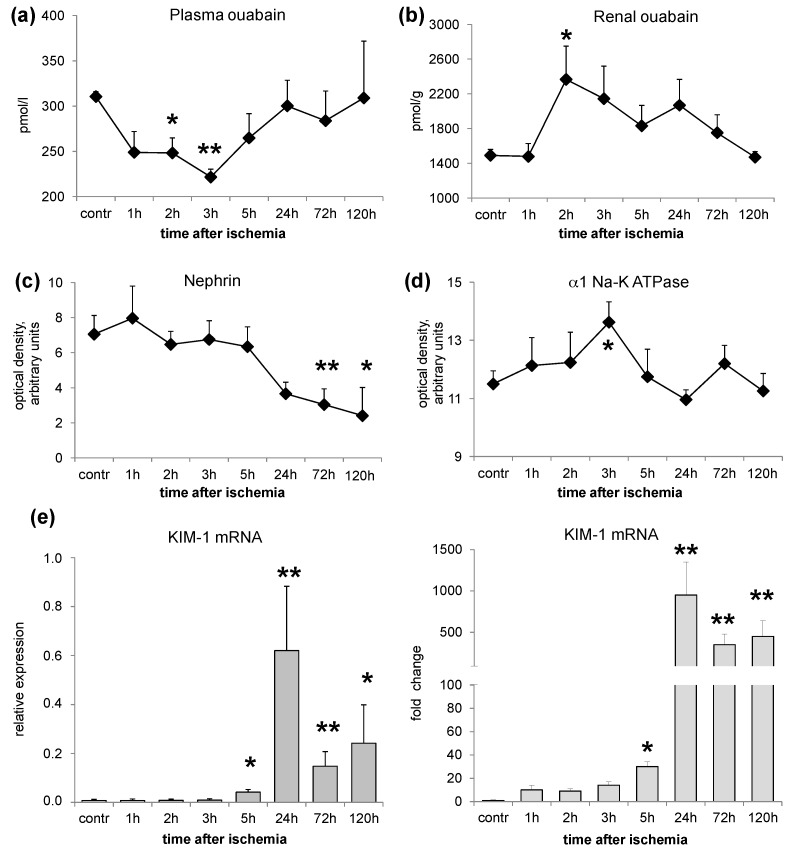
Time course of EO and protein marker changes after IRI (ischemia-reperfusion injury). Rats underwent right nephrectomy and ischemia was performed by clamping the left artery for 45 min. Reperfusion was followed after 1 to 120 h from the beginning of ischemia. At each time (1, 2, 3, 5, 24, 72, 120 h), rats (*n* = 3/8 per group, see Methods) were sacrificed and plasma and kidney were removed to measure endogenous ouabain (EO) levels and protein biomarkers. (**a**,**b**) Time course of EO changes in plasma of ischemic rats versus controls and in ischemic versus contra-lateral healthy kidney measured by radioimmunoassay (RIA); (**c**,**d**) Effect of ischemia-reperfusion on nephrin and α1 Na-K ATPase protein expression in the ischemic versus contra-lateral healthy kidney quantified by immunoblotting. Optical densities are expressed as arbitrary units; (**e**) Kidney-Injury-Molecule-1 (KIM-1) mRNA as relative expression in the ischemic kidney (**left**) and as fold change (**right**) versus contra-lateral healthy kidney determined by RT-PCR. Data are mean ± SEM, * *p* < 0.05; ** *p* < 0.01 vs. control.

EO was quantified by immunoassay in all plasma and kidney samples. In both specimen types, EO levels underwent fast and time-dependent changes. In plasma, EO significantly decreased 2–3 h after injury compared to controls and returned to normal levels starting from 24 h (Figure 1a). In parallel, when compared to the contra-lateral healthy kidney, the content of EO in the ischemic kidney significantly increased, with a peak effect 2 h after injury and completely returned to normal values 120 h later (Figure 1b).

**Figure 2:**
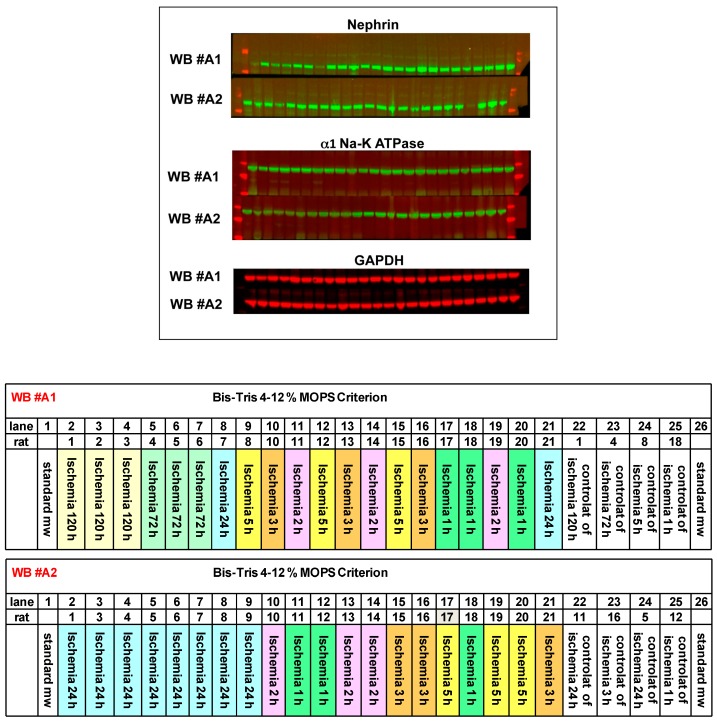
Representative images of the Western blots for nephrin and α1 Na-K ATPase of the time course experiment. GAPDH was used as loading control. Standards of molecular weight are shown on the left and right in red (for nephrin: 250, 150 kDa; for Na-K ATPase: 100, 75, 50 kDa). Two gels are shown: WB #A1 and WB #A2 (26 wells). Lane legends are shown in the lower panel, each lane representing one rat.

Glomerular and tubular markers were analyzed by immunoblotting or RT-PCR. In the ischemic kidney, the protein expression of nephrin—the specific glomerular podocyte marker—decreased time-dependently starting from 24 h after IRI and remained significantly lower throughout the experiment (120 h after injury), as compared to the contra-lateral healthy kidney (Figure 1c). Conversely, the tubular marker, α1 Na-K ATPase, modestly but significantly increased 3 h after IRI and returned to normal values 24 h after injury (Figure 1d). Figure 2 shows representative images of the Western blotting for nephrin and α1 Na-K ATPase. No significant variation of GAPDH levels over time was detected among rat groups (Figure 2).

The mRNA expression of KIM-1, a selective tubular kidney injury biomarker, resulted significantly upregulated 5 h after injury, it further increased over the contra-lateral healthy kidney after 24 h and remained highly expressed up to 120 h (Figure 1e).

These data suggest that IRI may cause time-dependent changes of plasma and renal EO levels and may induce glomerular and tubular alterations in the ischemic kidney.

### 2.2. Effect of Ouabain Infusion and Rostafuroxin Treatment on IRI-Induced Renal Damage 

In order to investigate whether high EO levels may directly contribute to worsen renal damage caused by ischemia-reperfusion maneuver, rats were subcutaneously infused with exogenous ouabain (30 µg/kg/day) (OHR rats) for eight weeks, while control rats received saline (control rats). After four weeks, OHR rats were divided into two groups receiving vehicle (group 2) or an oral treatment of 100 µg/kg/day rostafuroxin (group 4) for four weeks. Analogously, the control rats were allocated to two groups receiving vehicle (group 3) or 100 µg/kg/day rostafuroxin (group 5) for four weeks. At the end, all rats were subjected to renal ischemia and sacrificed after five days of reperfusion.

In OHR rats, SBP significantly increased versus control rats (153.4 ± 6.8 mmHg, *n* = 7, vs. 111.7 ± 2.8 mmHg, *n* = 7, *p* < 0.01). Rostafuroxin reduced SBP in OHR (−20 mmHg, *n* = 7, *p* < 0.01), but not in controls (+5 mmHg, *n* = 7), as previously shown [11].

IRI injury did not induce variations of body weight (BW) (Table 1), while the ischemic kidney wet weights, normalized by body weight, significantly increased versus contra-lateral healthy kidneys, both in controls and OHR rats (Table 1).

Tissue ouabain content similarly increased both in healthy and ischemic kidneys of OHR and controls (Table 2). Rostafuroxin did not induce any modification of body and kidney weight, measured before or after ischemia (Table 1), nor of plasma and kidney ouabain content, quantified in healthy or ischemic kidneys of OHR and control rats (Table 2).

**Figure 3:**
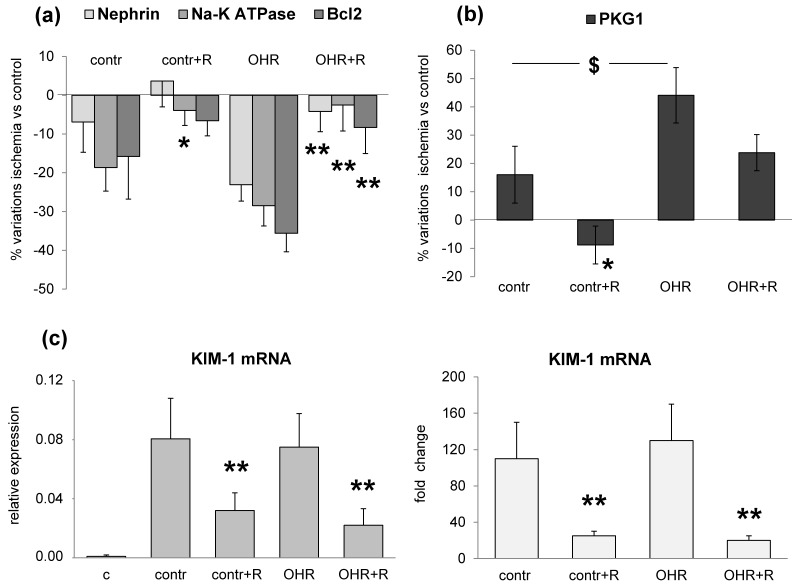
Effect of ouabain infusion and rostafuroxin treatment on IRI-induced alterations of renal markers. Ouabain was infused in rats at 30 µg/kg/day for eight weeks by osmotic mini-pumps (OHR rats). Controls received saline. After four weeks, a group of OHR and control rats received orally rostafuroxin at 100 µg/kg/day for four weeks, or vehicle. At the eighth week, all rats underwent ischemia and were sacrificed after five days. The expression of marker proteins was quantified in ischemic versus contra-lateral healthy kidney. Data are mean ± SEM, *n* = 8/13 rats per each group, see Methods. (**a**,**b**) Percent variation of protein expression in ischemic versus contra-lateral kidney for nephrin, α1 Na-K ATPase, Bcl2, and PKG1; (**c**) KIM-1 mRNA as relative expression in ischemic and contra-lateral kidney (**left**) and as fold change (**right**) in ischemic versus contra-lateral kidney quantified by RT-PCR. c, contra-lateral kidney; contr, control; R, rostafuroxin. * *p* < 0.05; ** *p* < 0.01 plus versus minus rostafuroxin; ^$^ *p* < 0.05 OHR versus contr.

**Figure 4:**
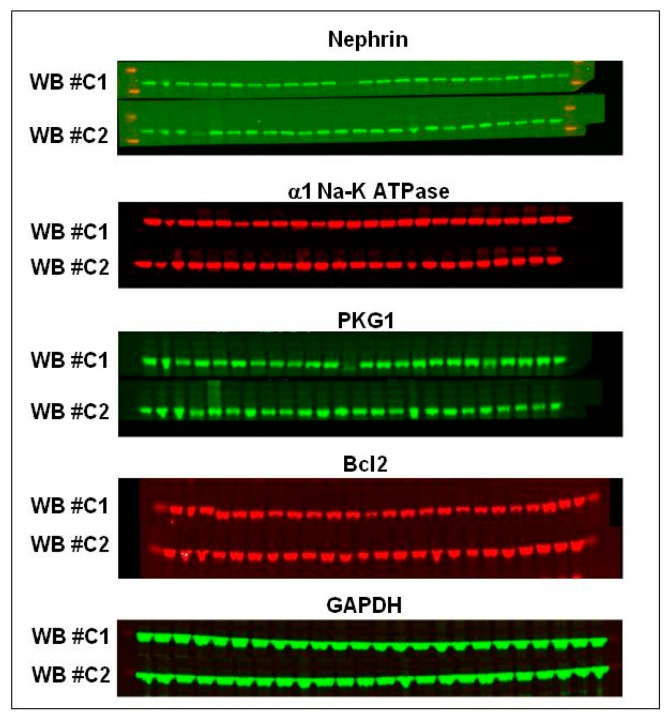

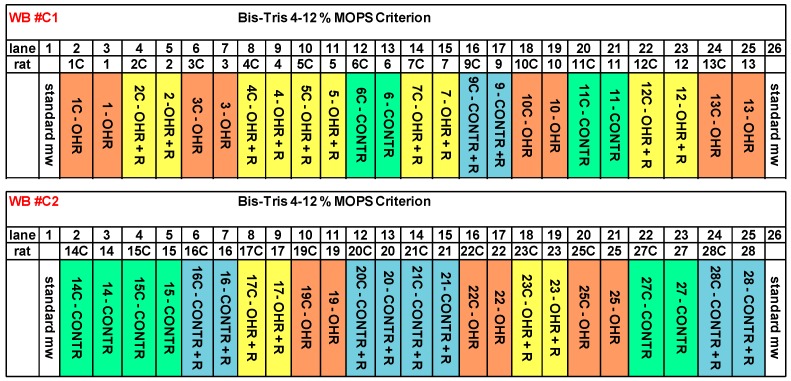
Representative images of the Western blots for nephrin, α1 Na-K ATPase, PKG1 and Bcl2 of the chronic experiment. GAPDH was used as loading control. Standards of molecular weight are shown on the left and right in red (MW: nephrin 185 kDa; Na-K ATPase 95 kDa; PKG1 75 kDa; Bcl2 26 kDa). Two gels are shown: WB #C1 and WB #C2 (26 wells). Lane legends are indicated in the lower panel, each lane representing one rat of the four groups: control, control + rostafuroxin (R), OHR, OHR + rostafuroxin (R).

Immunoblotting analysis revealed that ischemic kidneys from OHR rats displayed a significant downregulation of nephrin, α1 Na-K ATPase and Bcl2 and an upregulation of cGMP-dependent protein kinase-1 (PKG1) compared to their contra-lateral healthy kidneys (Figure 3a,b). The protein changes resulted greater in OHR than in normal rats, although statistical significance was achieved only for PKG1. Representative images of the Western blotting for nephrin, α1 Na-K ATPase, PKG1, and Bcl2 are shown in Figure 4. No significant variation of GAPDH level was observed among rat groups (Figure 4).

As shown in Figure 3c, KIM-1 mRNA increased in the ischemic kidney of control and OHR rats.

**Figure 5:**
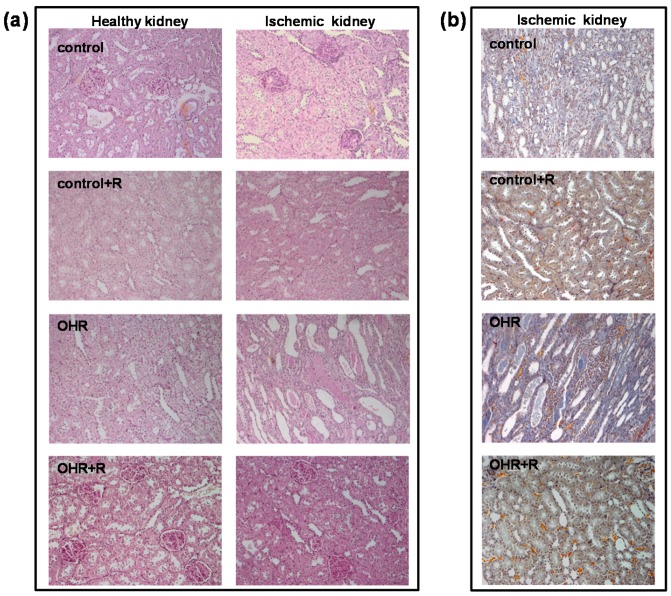
Representative PAS (**a**) and trichrome (**b**) staining images (magnification 200×) of renal sections from control and OHR rats. The histological analysis indicated the appearance of morphological alterations, including mesangial hypercellularity, mesangial matrix expansion, tubular casts, tubular dilation, interstitial infiltration, and interstitial fibrosis. These alterations were more evident in ischemic kidneys of OHR rats compared to controls and were reverted by rostafuroxin treatment. The evaluation of histological parameters was scored semi-quantitatively as shown in Table 3. The glomerular evaluation was performed on 30 glomeruli per section, indicating that it was modestly affected by ischemia and rostafuroxin (score in Table 3).

The histological evaluation demonstrated the appearance of morphological alterations, including mesangial hypercellularity, mesangial matrix expansion, tubular casts, tubular dilation, interstitial infiltration and interstitial fibrosis in ischemic kidneys mainly of OHR rats compared to controls, while segmental and global glomerulosclerosis was modestly different (Figure 5). The scores are shown in Table 3.

All these alterations were reverted by rostafuroxin treatment at a greater extent in OHR than in controls (Figure 3a–c and Figure 5).

## 3. Discussion

The present study provides the evidence that EO/ouabain may be implicated in the development of renal glomerular and tubular damage following IRI and that rostafuroxin may represent a novel pharmacological approach to prevent IRI-induced renal damage.

In a rat model of renal IRI, we studied the changes of EO both in plasma and in the renal tissue, together with glomerular and tubular markers of damage in two sets of experiments: normal rats and ouabain pre-infused rats. In the first set *(*group 1*)*, the time course of EO variations after IRI was investigated. The results indicate that acute warm ischemia caused a rapid decrease of plasma EO (within 2–3 h), accompanied by a parallel increase of renal EO content. Both alterations completely reverted after several hours (Figure 1). To reconcile these opposite changes of EO in plasma and in the renal tissue, it may be postulated that there is an increased capability of the ischemic renal tissue to bind EO. As ouabain is excreted by the kidney [12], a defect of renal EO excretion may also contribute to its raise in the renal tissue.

The increase of renal EO content parallels the appearance of glomerular podocyte alterations and renal tubule marker variations in the ischemic kidney (group 1, Figure 1). These consist in a time-dependent downregulation of nephrin associated with an early (3 h after IRI injury) increase of renal α1 Na-K ATPase, that progressively reduced after 24 h, and a persistent elevation of KIM-1 mRNA, starting from 24 h after IRI. The protein expression changes consequent to IRI injury may not imply the existence of a cause-effect relationship with renal EO increase. However, as observed in our previous studies [6,9] and here confirmed (Figure 1), ouabain, by itself, independently of blood pressure increases, may cause two separated types of changes: (1) nephrin downregulation, with a consequent damage of glomerular filtration barrier and associated proteinuria in rats, as described [6,8]; and (2) an upregulation of α1 Na-KATPase that has been associated with the activation of the Src-mediated signaling pathway responsible for the development of organ damage [9].

To verify whether a relationship between high renal EO content and glomerular and tubular markers may exist, we experimentally induced the increase of ouabain in plasma and kidney by infusing ouabain in rats (OHR), in comparison to controls (groups 2–5). After eight weeks, all rats were subjected to IRI injury and sacrificed five days later. Compared to controls, ouabain levels in OHR rats were tripled in plasma and increased 10-fold in both healthy and ischemic kidneys. 

In this setting, we documented the downregulation of nephrin, α1 Na-K ATPase and Bcl2 and the upregulation of PKG1 and KIM-1 in response to IRI injury (Figure 3). These alterations were present also in controls, even though they were more pronounced in ouabain-infused OHR rats. It is important to note that the increment of EO was similar in the pre- and post-ischemic kidney but the renal damage occurred only after ischemia. This may indicate that EO/ouabain-induced renal injury requires the presence of ischemia to develop. Furthermore, as shown previously [6,8] and here described, the prolonged infusion of ouabain in rats induces a significant increase of blood pressure, which may contribute to the worsening of renal damage in OHR rats versus controls not infused with ouabain. However, published data [6,8] have supported that nanomolar concentrations of ouabain may exert a direct effect on primary cultured podocytes through a mechanism that is independent of blood pressure variation. The ouabain-induced reduction of nephrin in podocytes is likely to underlie changes of the structural organization and function of the glomerular filtration barrier, responsible for podocyte foot process effacement and the progression to renal disease [6,8].

Consistently, evidence in literature has documented that the IRI maneuver may induce the downregulation of renal α1 Na-K ATPase, mainly in the proximal tubules, in association with the disruption of the actin-based cytoskeleton, which finally contributes to the deterioration of renal function [13]. Indeed, the actin network is of crucial importance for the mechanical stability of the cell and for protein trafficking [14]. Thus, an impairment of plasma membrane integrity may result in the activation of protein endocytosis, leading to the reduction of cell surface protein abundance, as occurs for α1 Na-K ATPase after IRI injury [15]. However, our data indicate a more complex effect of ouabain on α1 Na-K ATPase after IRI injury that seems to follow a bimodal trend. The enzyme appears upregulated, although modestly, at an early phase (2–3 h) after IRI, as observed in the acute experiment (group 1, Figure 1), and downregulated at later times (five days) after IRI, as documented in the chronic experiment (groups 2–5, Figure 3). The ouabain-mediated upregulation of α1 Na-K ATPase has been already commented [9] while the second phase of ouabain-mediated downregulation of Na-K ATPase after IRI may rely on the activation of an endocytotic process, possibly mediated via the over-expression of PKG1 [16]. Indeed, PKG1 is phosphorylated and activated by Src in a cGMP-dependent manner and consequently may favor the phosphorylation of α1 Na-K ATPase in Ser/Thr residues, inducing its internalization [16].

The novelty of the present study concerns the existence of a relationship between ouabain and renal glomerular and tubular damages after IRI and the evidence that such alterations are prevented by rostafuroxin. Therefore, the variety of previously suggested changes induced by IRI, including reactive oxygen species (ROS) production and micro-vascular inflammatory response, may have a common ground. The modest increase in focal and segmental glomerulosclerosis, here, may be anticipated by the need of a certain length of time to translate the molecular changes into morphological ones. 

The pharmacological treatment with rostafuroxin reverts IRI-induced renal glomerular and tubular lesions (Figure 3). The protective effect of this compound is also present in control rats with normal EO levels, but it appears more evident in OHR rats. Indeed, previous findings have indicated that rostafuroxin is a potent and safe drug, able to counteract hypertension and the associated cardiac and renal complications caused by EO in animal models [8,9,11,17]. More recently, a pharmacologic clinical trial has shown that rostafuroxin also normalizes blood pressure levels in patients carrying a selective genetic profile that includes genes encoding for enzymes controlling EO synthesis and transport and adducin genetic variants [10].

The data described here may be of particular relevance when translated into a clinical setting, where patients undergoing partial nephrectomy with warm renal ischemia may benefit from the pre-treatment with rostafuroxin. Whether this benefit is greater in carriers of the genetic profile mentioned above must be tested in a future clinical trial.

In conclusion, here we have provided the evidence that EO may be responsible for IRI-mediated renal injury and that high EO levels may contribute to a further deterioration of renal lesions following IRI. In rats, these alterations are antagonized by the pre-treatment with rostafuroxin, a new anti-hypertensive compound that antagonizes EO molecular and functional effects. Rostafuroxin effect has both a scientific and a clinical implication. In fact, it strengthens the role of EO as a unique triggering mechanism of a variety of changes associated to IRI and, on the other hand, it indicates that rostafuroxin may be the first-in-class drug able to limit/prevent the renal damage subsequent to IRI in patients. 

Of course, appropriate clinical studies are needed to translate these findings from animals to patients. Such studies have to include the measurement of plasma EO in patients with the appropriate genetic profile before partial nephrectomy to assess whether these measurements may furnish an estimation of the risk of renal damage.

As a limitation of the study, we cannot exclude that other potentially active substances, in addition to EO/ouabain, and other associated causes may contribute to IRI injury. However, since IRI-mediated glomerular and tubular damaging effects are blocked by rostafuroxin pre-treatment, we have to assume that the compound is able to counteract also the effects induced by these hypothetical substances.

## 4. Materials and Methods

### 4.1. Chemicals

The ouabain-antagonist rostafuroxin (PST2238; 17β-(3-furyl)-5β-androstan-3β,14β,17α-triol) has been synthesized in Prassis Sigma-tau Research Institute, Settimo Milanese, Milan, Italy and developed in Sigma-tau, Pomezia, Rome, Italy, and CVie Therapeutics, Taipei, Taiwan, China. The pharmacological characteristics of the compound are described elsewhere [11,17,18].

### 4.2. Ethics Statement

Animal use complied with the Institute for Animal Care guidelines, in compliance with Community guidelines 86/609 and with the Italian Law (DLI 16, 27 January 1992). Animals were housed in the pathogen-free facility with a 12–12 h light-dark cycle.

### 4.3. Rat Studies: Ischemia Surgical Procedure and Protocols

Under surgical anesthesia (1%–2% isofluorane, Sigma-Aldrich, Milan, Italy), laparotomy was performed through a midline incision. The right renal artery, vein, and ureter were legated and ipsilateral nephrectomy was performed. The left renal pedicle was exposed and occluded with a non-traumatic vascular clamp for 45 min and the left kidney was then reperfused. 

Sprague-Dawley rats from Charles River (Calco, Italy) underwent right nephrectomy followed by contra-lateral IRI. We studied the following conditions.

In the first study, we set up a time course experiment to evaluate EO and renal marker changes during the five days after ischemia (group 1). In this group, we used 38 male rats, 200–250 g, sacrificed after 1 h (*n* = 6), 2 h (*n* = 6), 3 h (*n* = 6), 5 h (*n* = 6), 24 h (one day, *n* = 8), 72 h (three days, *n* = 3), and 120 h (five days, *n* = 3) from the beginning of ischemia. Contra-lateral healthy kidneys were used as control (time zero, *n* = 8). At sacrifice, blood was collected and plasma obtained for endogenous EO measurement by RIA assay. Kidneys were snap-frozen in liquid nitrogen for EO content quantification, Real Time-PCR (KIM-1) and Western blot analyses (nephrin, α1 Na-K ATPase) for glomerular and renal tubular marker determination.

In a second study, we evaluated EO and renal marker changes after five days from ischemia in rats chronically infused with ouabain, or saline, and treated with rostafuroxin. We used 41 male rats divided in four groups (groups 2–5), as follows:

Group 2: 13 male rats, 120–150 g, were implanted with osmotic mini-pumps (Alzet, Charles River, Calco, Italy) delivering ouabain (30 μg/kg/day, Sigma-Aldrich) subcutaneously for eight weeks as described [11]. After four weeks, rats received the vehicle orally (0.5% Methocel, Sigma-Aldrich, OHR) for four weeks.

Group 3: 10 male rats, 120–150 g, were implanted with osmotic mini-pumps delivering sterile saline subcutaneously for eight weeks. After four weeks, rats received the vehicle orally (0.5% Methocel, control) for four weeks.

Group 4: 10 male rats, 120–150 g, were implanted with osmotic mini-pumps delivering ouabain, as in Group *2*. After four weeks, rats received rostafuroxin orally (100 μg/kg/day in 0.5% Methocel, OHR + R) for four weeks.

Group 5: 8 male rats, 120–150 g, were implanted with osmotic mini-pumps delivering sterile saline, as in Group 3. After four weeks, rats received rostafuroxin orally (100 μg/kg/day in 0.5% Methocel, control + R) for four weeks.

At the eighth week, all rats from groups 2–5 were subjected to renal ischemia and sacrificed after five days. Plasma and kidneys were collected and snap-frozen for further molecular and histological analyses.

The following parameters were measured in rats from groups 2–5: weekly systolic blood pressure (SBP) and heart rate (HR) by tail-cuff plethysmography (Model 29, Blood Pressure Meter/Amplifier IITC, Inc., Woodland Island, CA, USA); plasma and kidney EO levels measured by RIA assay; quantification of glomerular podocyte and renal tubular protein expression (nephrin, α1 Na-K ATPase, Bcl2, PKG1) by immunoblotting and KIM-1 by Real Time-PCR; histopathological analysis.

### 4.4. Plasma and Tissue EO/Ouabain Determination

EO/ouabain was extracted with methanol from rat plasma and kidney and was quantified by a radioimmunoassay by using a specific anti-ouabain antibody, as described [19]. Although the precise chemical structure of EO has not been fully identified, additional analysis by liquid chromatography-mass spectrometry has suggested that EO may be a structurally related isomer of ouabain [20]. We are aware of this limitation and in the present paper we assimilate EO with ouabain.

### 4.5. Histological Analysis

Periodic acid–Schiff, PAS (Sigma-Aldrich), and Acid Fuchsin Orange-G, AFOG (Sigma-Aldrich), trichrome stainings were performed according to standard techniques. Histological parameters, including mesangial hypercellularity, mesangial matrix expansion, tubular casts, tubular atrophy, interstitial infiltration, and interstitial fibrosis, were scored semi-quantitatively from − to +++ (−, none; +, mild; ++, moderate; +++, severe) by two independent observers, not aware of rat treatment. Glomerular evaluation was performed on 30 glomeruli per section and the presence of segmental and global glomerulosclerosis was quantified. The sections were examined with a Zeiss Axioskop 40 microscope (Zeiss, Thornwood, NY, USA).

### 4.6. Real Time-PCR

Total RNA was prepared from isolated kidney by using ultra-pure TRIzol reagent (GIBCO-BRL, Grand Island, NY, USA). The RNA (1 μg) was retro-transcripted using a specific kit (ROCHE, Monza, Italy). KIM-1 and GAPDH, specific TaqMan probes, were from Applied Biosystems (Applied Biosystems Inc., Foster City, CA, USA). Each sample was run in triplicate. The 2^–ΔΔ*C*t^ method was used to quantify the relative expression and the comparative threshold cycle method was used to quantify fold increase (2^–ΔΔ*C*t^) compared to controls.

### 4.7. Western Blot Analysis

Renal homogenates were separated by SDS-PAGE electrophoresis, blotted on nitrocellulose membrane (Bio-Rad, Hercules, CA, USA), and incubated with primary antibodies, followed by 1 h incubation with fluorescent secondary antibodies (Alexa Fluor, 680 nm). The Western blotting was analyzed and quantified by Odyssey Infrared Imaging Detection System (LICOR Biosciences, Lincoln, NE, USA). The optical densities were expressed as arbitrary units. Antibodies: anti-nephrin (Progen, Heidelberg, Germany); anti-actin (Sigma-Aldrich); anti-α1 Na-K ATPase (Hybridoma Bank, clone a6F, Iowa City, IA, USA); anti-PKG1 (Cell Signaling Technology, Danvers, MA, USA); anti-Bcl2 (Santa Cruz Biotechnology, CA, USA); anti-GAPDH (Abcam, Cambridge, UK).

### 4.8. Statistical Analysis

Data are reported as mean ± SEM. The statistical significance was measured by ANOVA, *p* < 0.05 was considered statistically significant.

## Figures and Tables

**Table 1:** Mean body weight and kidney weight of rats from the chronic experiment.

Chronic Experiment				
	Body Weight (BW, g)		Kidney Weight/BW	
	PRE-Ischemia	POST-Ischemia 5 Days	PRE-Ischemia	POST-Ischema 5 Days
Control	465 ± 8.4	433 ± 13.9	0.391 ± 0.012	0.555 ± 0.054 *
Control + rostafuroxin	455 ± 7.2	440 ± 9.4	0.380 ± 0.011	0.460 ± 0.048
OHR	464 ± 8.7	453 ± 9.8	0.402 ± 0.011	0.524 ± 0.046 *
OHR + rostafuroxin	456 ± 11.5	428 ± 12.2	0.364 ± 0.038	0.493 ± 0.043 *

Control, *n* = 10; control + rostafuroxin, *n* = 8; ouabain hypertensive rats (OHR), *n* = 13; OHR + rostafuroxin, *n* = 10. Data are mean ± SEM. * *p* < 0.05 post- vs. pre-ischemia.

**Table 2:** Plasma and kidney ouabain content of rats from the chronic experiment.

Chronic Experiment			
	Plasma Ouabain (nM)	Kidney Ouabain (nmol/g)	
	POST-Ischemia 5 Days	PRE-Ischemia	POST-Ischemia 5 Days
Control	0.193 ± 0.029	2.44 ± 0.128	2.29 ± 0.101
Control + rostafuroxin	0.156 ± 0.011	2.47 ± 0.109	2.31 ± 0.115
OHR	0.757 ± 0.072 **	26.04 ± 1.9 **	27.03 ± 1.033 **
OHR + rostafuroxin	0.753 ± 0.077 **	34.61 ± 4.42 **	30.49 ± 2.53 **

Chronic experiment: Control, *n* = 10; control + rostafuroxin, *n* = 8; OHR, *n* = 13; OHR + rostafuroxin, *n* = 10. Data are mean ± SEM. ** *p* < 0.01 ouabain-infused rats vs. controls.

**Table 3:** Histological evaluation on contra-lateral healthy and ischemic kidney of control and OHR rats.

Rat Groups	Healthy vs. Ischemic	Mesangial Hypercellularity	Mesangial Matrix Expansion	Focal/Segment Glomerulo-Sclerosis	Tubular Casts	Tubular Damage	Interstitial Inflammation	Interstitial Fibrosis
Control	*healthy*	+	+	2/30	+/−	+	+	+
	*ischemic*	++	+	6/30	++	++	++	++
Control + R	*healthy*	++	++	4/30	+	+	+	+
	*ischemic*	++	++	6/30	+	+	++	+
OHR	*healthy*	+	++	4/30	+	+	+	++
	*ischemic*	+	+	5/30	+++	+++	+++	++
OHR+R	*healthy*	++	+	5/30	−	+	+	+
	*ischemic*	+	+	6/30	+	+	+	+

Histological evaluation was performed on two rats per each group and was scored semi-quantitatively from − to +++ (−, none; +, mild; ++, moderate; +++, severe). Glomerular evaluation was performed on 30 glomeruli per section and the presence of segmental and global glomerulosclerosis was quantified. Representative histological images are shown in Figure 5. R = Rostafuroxin.
